# The role of tumour microenvironment: a new vision for cholangiocarcinoma

**DOI:** 10.1111/jcmm.13953

**Published:** 2018-11-05

**Authors:** Ziyan Chen, Pengyi Guo, Xiaozai Xie, Haitao Yu, Yi Wang, Gang Chen

**Affiliations:** ^1^ Department of Hepatobiliary Surgery The First Affiliated Hospital Wenzhou Medical University Wenzhou China; ^2^ Environmental and Public Health School of Wenzhou Medical University Wenzhou China

**Keywords:** cholangiocarcinoma, tumor microenvironment, tumor stromal cells

## Abstract

Cholangiocarcinoma (CCA) is a relatively rare malignant and lethal tumour derived from bile duct epithelium and the morbidity is now increasing worldwide. This disease is difficult to diagnose at its inchoate stage and has poor prognosis. Therefore, a clear understanding of pathogenesis and major influencing factors is the key to develop effective therapeutic methods for CCA. In previous studies, canonical correlation analysis has demonstrated that tumour microenvironment plays an intricate role in the progression of various types of cancers including CCA. CCA tumour microenvironment is a dynamic environment consisting of authoritative tumour stromal cells and extracellular matrix where tumour stromal cells and cancer cells can thrive. CCA stromal cells include immune and non‐immune cells, such as inflammatory cells, endothelial cells, fibroblasts, and macrophages. Likewise, CCA tumour microenvironment contains abundant proliferative factors and can significantly impact the behaviour of cancer cells. Through abominably intricate interactions with CCA cells, CCA tumour microenvironment plays an important role in promoting tumour proliferation, accelerating neovascularization, facilitating tumour invasion, and preventing tumour cells from organismal immune reactions and apoptosis. This review summarizes the recent research progress regarding the connection between tumour behaviours and tumour stromal cells in CCA, as well as the mechanism underlying the effect of tumour stromal cells on the growth of CCA. A thorough understanding of the relationship between CCA and tumour stromal cells can shed some light on the development of new therapeutic methods for treating CCA.

## 1 INTRODUCTION

Cholangiocarcinoma (CCA), the second most common hepatic carcinoma, is an epithelial malignant tumour in the intrahepatic and extrahepatic bile ducts from hepatic hilar region to the lower portion of the common bile duct. According to its anatomical location in the biliary tree, CCA can be divided into intrahepatic, perihilar, and distal CCA, with more than 90% in the extrahepatic bile duct (50% perihilar CCA and 42% distal CCA) and less than 10% within liver.^1^ It often occurs in the background of chronic liver inflammation and shows correlations with liver cirrhosis, hepatitis virus infection, primary sclerosing cholangitis, liver fluke infection, and other related disease.^2^, ^3^, ^4^ CCA is a devastating and aggressive disease that has dismal outcomes due to its late clinical presentation and stubborn resistance to chemotherapy. Surgical treatment is currently the first clinical choice for treating CCA,^1^ but the treatment efficiency is low, yielding a poor prognosis and a low 5‐year survival rate of 23.7% and the recurrence rate is high.^5^ In accordance with previous research, tumour cells are dedicated to build their own favourable context by incorporating extracellular matrix, stromal cells that secret tumour‐related mediators, and tumour angiogenesis that provides more blood supply for tumour growth. Hence, tumour microenvironment promotes proliferation of tumour cells, assists tumours to escape from anti‐tumour immune reactions, and enhances the resistance of tumour cells to treatment.^6^ A study by Leyva‐Illades et al. showed that CCA cells can promote formation of surrounding connective tissue under the support from an abundant tumour microenvironment, and this process contributes prominently to therapeutic resistance of CCA.^7^ Maurizio Romano and colleagues reported that the angiogenesis, metastasis, invasion, and occurrence of CCA are closely related to the tumour microenvironment and can be regulated by the interaction between CCA stem cells (a component of CCA stromal cells) and tumour microenvironment.^8^

## 2 MOLECULAR MECHANISMS OF CHOLANGIOCARCINOMA

**Figure 1:**
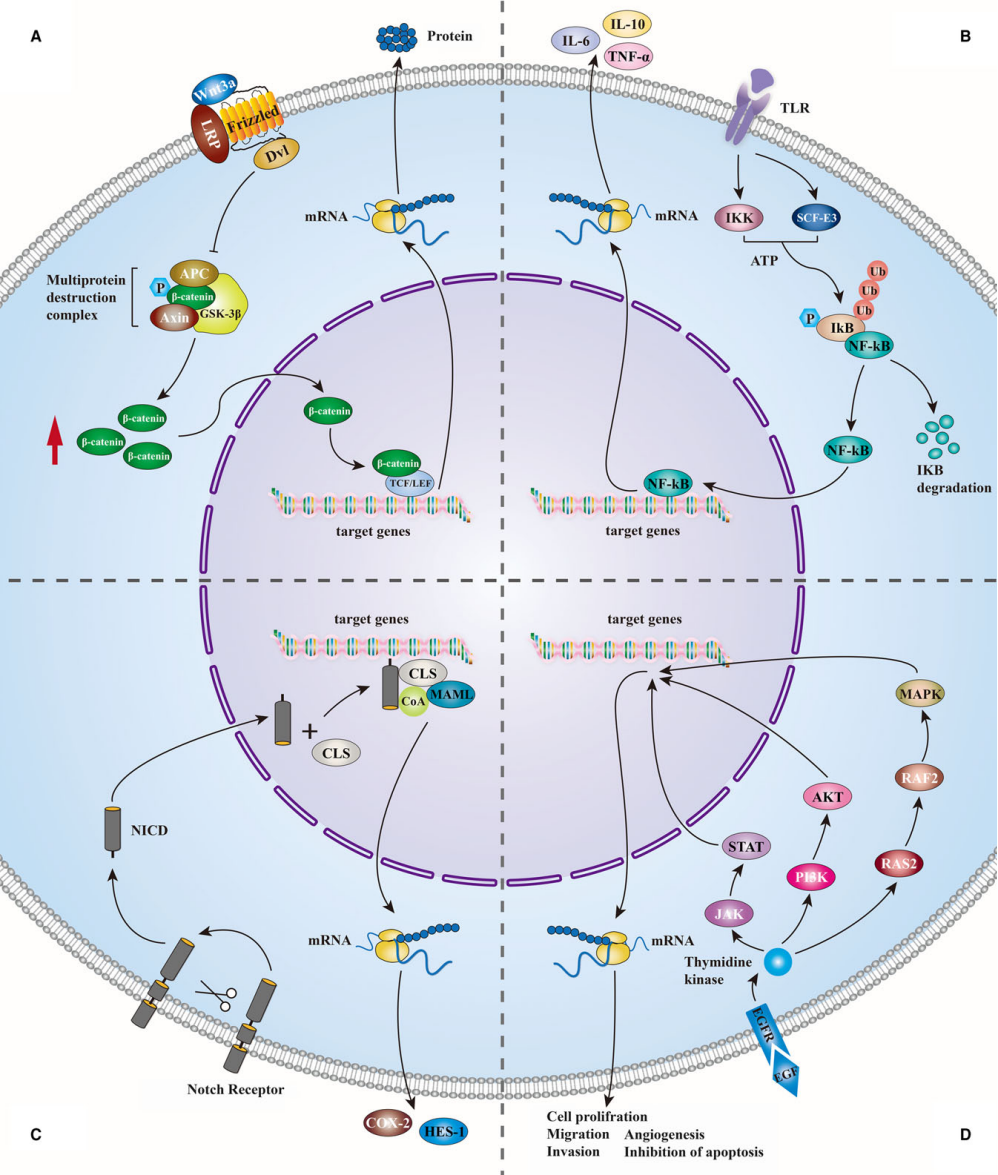
The pathways participate in tumourigenesis of CCA. A, Extracellular WNT glycoproteins appear and bind to Frizzled receptors and the co‐receptors LRP5 (low‐density lipoprotein receptor‐related protein 5) and LRP6, DVL inhibit the destruction complex that made of APC, GSK3β and AXIN and results in the accumulation of β‐catenin in cell cytoplasm. β‐catenin enters nucleus and combines with TCF/LEF transcription factors to regulate the expression of target genes. Then, influencing the CCA. B, NF‐kB integrates with inhibiting factor IkB in the stationary state. After IkBs are phosphorylated by IKK or ubiquitnated by SCF‐E3 and degraded by protease, NF‐kB is activated and entering nucleus to induce the transcription of target genes. C, DSL family bind to notch receptors and stimulate the notch protein shear. And the generated NICD turns into nucleus to combine with transcription factor CLS. The compound activates the expression of target genes to induce CCA (D) EGFRs combine with EGFR ligands to activate protein kinases. Thymidine kinase is phosphorylated to stimulate Ras2/Raf2/MAPK signalling pathway, PI3K/AKT pathway and JAK/STAT pathway that play important roles in CCA carcinogenesis

**Figure 2:**
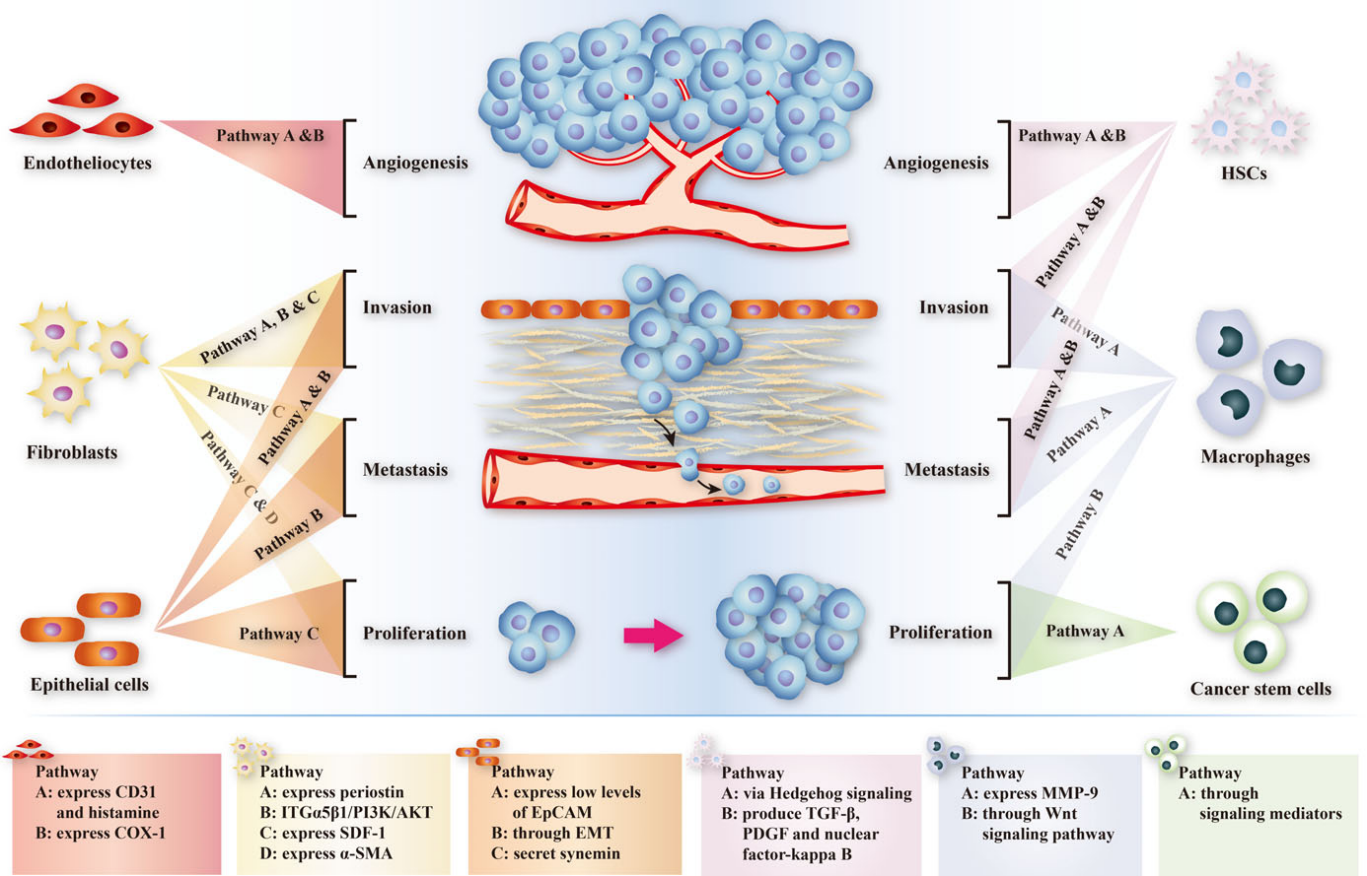
Work model of the impact of tumour stromal cells on CCA. Endothelial cells promote CCA angiogenesis through pathway A (release inflammatory cytokines) and B (express COX‐1 protein). Fibroblasts induce CCA invasion via pathway A (produce periostin), B (ITGα5β1/PI3K/AKT pathway) and C (express SDF‐1), stimulate CCA metastasis through pathway A (express SDF‐1) and promote proliferation of CCA by means of pathway A (express α‐SMA) and B (produce SDF‐1). Epithelial cells promote CCA invasion via pathway A (express low level of EpCAM) and B (epithelial‐mesenchymal transition), induce CCA proliferation through pathway A (secret synemin) and stimulate CCA metastasis by means of pathway A (epithelial‐mesenchymal transition). HSCs induce the angiogenesis, invasion and metastasis via pathway A (Hedgehog signalling) and B (produce TGF‐β, PDGF and nuclear factor‐Kappa B). Macrophages stimulate invasion and metastasis of CCA via pathway A (express MMP‐9), induce CCA proliferation through Wnt signalling pathway. Cancer stem cells induce CCA proliferation by means of pathway A (produce signalling mediators)

Significant progress has been made in revealing molecular mechanisms underlying the pathogenesis of CCA, contributing to the accurate targeted therapies for patients. Wnt/β‐catenin signalling pathway is one of the significant signalling networks that induces tumourigenesis and tumour progression in CCA.^9^, ^10^ WNT protein, a type of secreted glycoprotein expressed by Wnt gene, binds to the Frizzled family receptors on cell membrane to activate Dishevelled (DVL), which then inhibits the activity of the complex made up of axin, adenomatous polyposis coli tumour suppressor protein (APC) and glycogen synthase kinase (GSK)‐3β and suppresses β‐catenin phosphorylation. The accumulated unphosphorylated β‐catenin in the cytoplasm can enter the nucleus and combine with TCF/LEF transcription factors to regulate the expression of oncogenes involved in CCA tumourigenesis, proliferation, and drug resistance like cyclin D1 and c‐Myc.^11^, ^12^, ^13^ In support of the notion that Wnt signalling promotes CCA tumourigenesis, Liu et al. confirmed that activated GSK‐3β acts as an important mediator in the inhibition of CCA cells based on experimental studies.^14^ In addition, a number of chemicals were found to have anti‐CCA effects via suppressing the Wnt/β‐catenin signalling pathway, indirectly confirming the role of this pathway in tumourigenesis of CCA.^9^, ^15^ Another crucial signalling pathway contributing to CCA is nuclear factor kappa B (NF‐κB) signalling pathway. NF‐κB, which binds to its inhibitor IκB in unstimulated cells, is activated when IκB is phosphorylated by the protein kinase of IκBs (IKK) or ubiquitnated by SCF‐E3 and degraded by protease. Activated NF‐κB enters the nucleus and binds to DNA to induce the transcription of target genes, thus modulating the growth and development of CCA.^16^ Performed with a series of in vitro experiments, Srikoon et al. revealed that the inhibition of NF‐κB signalling pathway impedes CCA metastasis and migration via suppressing the transcription of its target genes that express intercellular cell adhesion molecules in CCA cell lines.^17^ What's more, a growing number of substances have been found to inhibit CCA by blocking NF‐κB signalling pathway like Magnolol,^18^ Berberine,^19^ Caffeic acid phenethyl ester,^20^ and beta‐eudesmo.^21^ The above evidence indirectly demonstrates the importance of NF‐κB signalling pathway in the development of CCA. The notch signalling pathway also plays an important role in CCA progression. Binding of notch ligands and receptors induce the shear of notch protein, and then the generated notch intracellular domain (NICD) enters the nucleus to form a complex with transcription factor CLS (a kind of DNA‐binding protein),^22^ which activates the expression of CCA‐inducing target gene.^23^ DSL family members act as notch ligands, of which Jagged 1 is one of the most significant CCA‐associated notch ligands,^24^ and Notch2 receptors have a close relationship with CCA.^25^ The activation of notch signalling together with the inactivation of tumour suppressive gene p53 induced CCA tumourigenesis, growth,^26^ aggressiveness, and malignant transformation. Claperon et al. found that the epidermal growth factor receptor (EGFR) pathway also contributes to the invasion, metastasis, and development of CCA.^27^ EGFRs on cell membrane can bind with their ligands such as transforming growth factor α (TGF‐α) to activate protein kinases to promote CCA growth and the overexpression of EGFRs can be effective prognostic factors for intrahepatic CCA.^28^ In addition, activated EGFRs can regulate a few intracellular signalling pathways to influence cholangiocarcinogenesis. The downstream pathways contain the Ras2/Raf2/mitogen activated protein kinase (MAPK) signalling pathway, phosphatidyl inositol 3‐kinase/threonine kinase (PI3K/AKT) signalling pathway and signal transducers, and activator of transcription (STAT) signalling pathway.^29^ The molecules involved in the pathways mentioned above are mostly released from or affected by the surrounding tumour microenvironment (Figures 1 and 2).

## 3 INVOLVEMENT OF MICROENVIRONMENTAL FACTORS IN CCA OCCVURRENCE AND MALIGNANT BEHAVIOURS

CCA tumour microenvironment is created by the CCA itself or CCA‐induced reactions and in turn remarkably affects tumour behaviour. This dynamic environment consists of authoritative CCA stromal cells and extracellular matrix where CCA stromal cells and CCA cells can thrive. CCA‐associated stromal cells include two categories, immune cells and non‐immune cells. The former includes of tumour‐infiltrating immunocytes (eg regulatory T cells, regulatory B cells, and tumour‐associated macrophages), tumour associated fibroblasts that promote proliferation of tumour cells, and inflammatory cells that might contribute to tumourigenesis.^30^ Non‐immune cells mainly include myeloid‐derived suppressor cells (MDSCs) that construct immunosuppressive environment via synthesizing immunity suppressive factors. For example, MDSCs can produce inducible nitric oxide synthase (iNOS), arginase (ARG)‐1 and reactive oxygen species (ROS), which blocks the interferon (IFN)‐γ pathway and thus weakens or restrains anti‐tumour activity of immunocytes (in particular T cells and nature killer cells).^31^ CCA stromal cells can modulate the state and activity of CCA cells and contribute to immunosuppression, tumour invasion and chemo‐resistance.^30^ Besides, hepatic stellate cells (HSCs), one type of CCA peculiar stromal cells, can produce a large amount of extracellular matrix (ECM), thus promoting the proliferation and invasion of CCA cells and enhancing angiogenesis through activation of Hedgehog (HH) signalling.^32^

### 3.1 Hypoxia

Because of the irregular vascularization, rapidly proliferating tumour cells in some regions need to tolerate hypoxia that induces transform from aerobic oxidation glycolysis in cancer cells. A large proportion of influences of hypoxia for tumour growth involve hypoxia inducible factor (HIF) which is composed of two subunits (HIFα and HIFβ). Hypoxia induces the accumulation of HIFs by suppressing the hydroxylation of the subunit of HIF. Subsequently, HIFs interact with the coactivator p300 and bind to the promoters of HIF target genes to induce genetic instability and enhance the rate of gene mutations associated with metastasis, invasion, angiogenesis, progression, and the treatment‐resistant of tumour.^33^, ^34^ Through manipulating the interaction between HIFs and coactivator p300/CBP, Kung et al. revealed that the inhibition of hypoxia‐inducible transcription disturbs tumour progression in xenograft mouse colon and breast tumour models.^35^ As for CCA, hypoxia modulates the genes expression to influence the production of proteins which are associated with cell cycle, apoptosis, cellular movement in CCA cells. For instance, hypoxia upregulates proteins participating in tumour proliferation, such as trefoil factor‐1 (TFF1), metalloprotease 12 (ADAM12), integrin‐alpha 5 (ITGA5) and baculoviral IAP repeat‐containing 5 (BIRC5/surviving), and downregulates factors related to cell adhesion, such as uridine 5′‐monophosphate synthase (UMPS) and S100 calcium binding protein P (S100P), thus promoting CCA invasion and proliferation.^36^ And the invasion of CCA also can be enhanced by hypoxia through hepatocyte growth factor receptor (Met)/extracellular signalregulated kinase (ERK) pathway.^37^ In addition, hypoxia induces expressions of hypoxic‐responsive proteins (especially HIF‐1α), which are related to low CCA survival rate and poor prognosis of CCA.^38^, ^39^

### 3.2 Exosomes

Exosome, a crucial part of tumour microenvironment which contains microRNAs, DNA fragments, and proteins, serve as vital communicators among cancer cells, immunocytes, and tumour microenvironment to modulate tumour growth.^40^ Performed with cell transfection, western blotting (WB) analysis, and splenocyte proliferation assay, Rong et al. proved that exosomes derived from tumour cells can promote breast cancer progression through releasing abundant immunosuppressive cytokines (TGF‐β) to suppress immune reactions.^41^ Tumour microenvironment derived exosomes, which are secreted by tumour stromal cells (principally tumour associated fibroblasts), promote tumour growth by providing cancer cells with the substance needed for metabolism under a nutrient‐deficient environment.^42^ As for CCA, exosomes play an important role in regulating tumour growth. CCA‐derived exosomes assist CCA cells to escape from immune attack by preventing cytokine‐induced killer (CIK) cells from producing tumour necrosis factor (TNF)‐α and perforin that is critical in the anticancer activities.^43^ Development and metastasis of CCA is also modulated by CCA‐derived exosomes which can transfer oncogenic proteins, and oncogenic proteins can accelerate tumour progression by inducing β‐catenin and reducing E‐cadherin expressions in CCA cells.^44^

### 3.3 Chronic inflammation and cytokines

An increasing number of research findings have confirmed Rudolf Virchow's postulation that tumours evolve from chronic inflammation.^45^ As a very common and important basic pathological process, moderate inflammation alleviates the impairment from pathogenic factors, and chronic inflammation might induce tumourigenesis and protect cancer cells from immune attack via inducing dendritic cells to produce immunosuppressive factor indoleamine 2,3 dioxygenase (IDO).^46^ Chronic inflammation promotes tumourigenesis through supplying bioactive molecules such as inflammatory cytokines. Inflammatory cytokines, the transfer medium of signals between cells, play an essential role in intercellular signalling network not only in normal tissues but also in cancerous tissues besides CCA. Inflammation‐associated cytokines (including interleukin‐1β, IFN‐γ, and TNF‐α) in tumour microenvironment induce CCA cells to express iNOS that catalyses the production of NO. Additionally, NO oxidize DNA and suppress the DNA repair enzymes activity to induce carcinogenesis and initiation of CCA.^47^

#### 3.3.1 Transforming growth factor‐β

TGF‐β, a vital inflammatory cytokine, binds to TGF‐β receptors to active the downstream signalling of TGF‐β signalling. The activation of TGF‐β signalling decreases epithelial cadherin (E‐cadherin) and increases neural cadherin (N‐cadherin) through epithelial‐to‐mesenchymal transition (EMT) to promote the metastasis, invasion, and progression of CCA and other tumours.^48^, ^49^, ^50^ Blocking the TGF‐β signal pathway can promote the apoptosis of the CCA cells, which makes targeting the TGF‐β signal pathway has potential effect on the treatment of CCA.^51^ However, David et al. found that TGF‐β signalling serves as not only a tumour promotor but also a tumour suppressing factor through transforming TGF‐β‐induced Sox4 from an inducer of the tumourigenesis to a suppressor of tumour proliferation in pancreatic ductal adenocarcinoma.^52^

#### 3.3.2 Vascular endothelial growth factors

As specific growth factors of vascular epithelial cells, the fundamental function of vascular endothelial growth factors (VEGFs) is to induce angiogenesis. According to the experimental observation, Vaeteewoottacharn et al. demonstrated that VEGFs were highly expressed in CCA cells. And anti‐VEGFs treatment plays an important role in the treatment of CCA.^53^ VEGF‐D regulates the activities of stromal cells and tumour cells in tumour lymphatic metastasis by binding to lymphatic growth factor receptor (VEGFR)‐3.^54^ Similarly, VEGF‐C induces lymphatic invasion and metastasis in intrahepatic CCA by the preexisting lymphatic vessels in the tumour margin.^55^ Jinqiang Zhang and colleagues verified that miR‐101 restrains transcription and translation of VEGF genes and the low expression of VEGF inhibits CCA angiogenesis.^56^

#### 3.3.3 Other cytokines and chemokines

Besides TGF‐β and VEGFs mentioned above, TNF‐α, interleukin (IL)‐6, IL‐10, IFN‐γ, platelet derived growth factor (PDGF), and basic fibroblast growth factor (bFGF) regulate tumour differentiation by communicating with tumour stromal cells.^57^ CXCL9, CXCL10, CXCL13, CCL17, CCL19 can attract relevant immunocytes (macrophages, dendritic cells, MDSCs, mast cells, natural killer cells, B cells, and T cells) to tumourigenic regions, and then the interactions between chemokines and tumour cells will affect the control efficiency of immunity and induce the loss of coordination between immune contexture and cancer cells.^58^ TNF‐α and IFN‐γ upregulate the expression of chemokine receptors (particularly CCR5) in CCA cells and induce the production of CCL5 in mesenchymal stem cells (MSCs). And the CCL5/CCR5 axis promotes the metastasis and growth of CCA by Akt/NF‐κB signalling which enhances the expression of metal matrix proteinase (MMP).^59^ As an upstream activator of STAT3, IL‐6 induces malignant transformation and metastasis of CCA.^60^ In addition, TNF‐α can regulate EMT to accelerate CCA invasion.^61^ Taken together, interleukin, growth factors, chemokines, proangiogenic factors, and modifying enzymes secreted by CCA stromal cells establish a network of cytokines to impact CCA progression.

### 3.4 Fibroblasts

Through genetic lineage tracing and transplantation assays, Rinkevich et al. confirmed that fibroblasts are related to the formation of cancer stroma.^62^ Fibroblasts are recruited to the CCA tumourigenic region and transformed into “active fibroblasts” or CCA associated fibroblasts which make up a large proportion of CCA stromal cells. CCA associated fibroblasts can induce the proliferation and invasion of CCA by producing alpha‐smooth actin (α‐SMA), fibroblast‐specific protein‐1, fibroblast activation protein, and PDGFR‐β.^63^ PDGF‐D secreted by CCA cells binds to the PDGF receptor (PDGFR β) expressed by CCA associated fibroblasts to stimulate the migration of CCA associated fibroblasts and recruit CCA associated fibroblasts via activating Rac1 and Cdc42 Rho GTPases and c‐Jun N‐terminal kinase (JNK) in fibroblasts.^64^ Consequently, CCA cells can be surrounded by a wide array of CCA associated fibroblasts, which accelerate CCA fibrogenesis and progression. In addition, a higher level α‐SMA expressed by CCA associated fibroblasts can promote the growth of CCA and proliferation of biliary epithelial cells and shorten the survival time of CCA patients.^65^ Periostin (PN) over‐expressed in CCA associated fibroblasts activates ITGα5β1(a receptor on the membrane of CCA cells) and promotes the invasion of CCA through PI3K/AKT pathway.^66^ And PN can be considered as a promising biomarker for tumour progression and poor prognosis of CCA.^67^ Moreover, CCA associated fibroblasts express SDF‐1 to induce the invasion and migration of tumour by SDF‐1/CXCR4 interaction in CCA and this process can be enhanced by TNF‐α through increasing the expression of CXCR4 on CCA cells.^68^ SDF‐1 expressed by CCA associated fibroblasts and TGF‐β 1 secreted from CCA cells is involved in invasion, migration, and proliferation of CCA cells.^69^

### 3.5 Inflammatory cells

Inflammatory cells include immunocytes, such as lymphocytes, granular leukocytes, and monocytes, as well as mast cells and endothelial cells that originally exist in tissues. Inflammatory mediators (eg iNOS, monocyte chemotactic protein 1) released from inflammatory cells and tumour cells impact the processes of inflammation and tumourigenesis and the process of producing and releasing inflammatory mediators is mainly regulated by MAPK pathway.^70^ Chronic inflammation, developed mostly following mechanical damage, parasite secretion,^71^ and immunopathology, is one of the most significant factors inducing hepatic fibrosis and cirrhosis and promoting occurrence of CCA.^72^ STAT family, particularly STAT3, which can be activated by inflammation and highly expressed in the inflammatorily lesioned tissue, is associated with CCA carcinogenesis and prognosis.^73^ High expression of activation‐induced cytidine deaminase (AID) induced by pro‐inflammation cytokine mediates the progression from bile duct inflammation to CCA carcinogenesis via inducing genetic aberrations.^74^ Taken together, inflammation and stromal cells particularly inflammatory cells that participate in inflammation response play what a crucial role in the initiation and development of CCA from the above‐mentioned content.

#### 3.5.1 Macrophages

Tumour associated macrophages (TAMs) derived from monocytes in the blood are recruited by cytokines and chemokines secreted by cancer cells to constitute a crucial element of tumour stromal cells.^75^ TAMs provide immune escape for cancer cells by expressing immunosuppressive cytokines (IL‐10) which induces T cell dysfunction^76^ and promote tumour invasion and migration by secreting extracellular matrix remodeling enzymes (MMP‐1, MMP‐3, MMP‐10).^77^ Under the action of colony‐stimulating factor (CSF), TAMs contribute to the enhanced invasion, angiogenesis, and immunoregulation of non‐small cell lung cancer.^78^ Performed with an Ov‐induced hamster CCA model, Thanee et al. revealed that alteration of TAMs characterizes early CCA and TAMs are key players in promoting progression and metastasis of CCA.^79^ TAMs express matrix metalloproteinase‐9 (MMP‐9) to degrade the extracellular matrix that contributes to the metastasis, invasion and low survival rate of CCA.^80^ By analyzing surgically resected tumour tissues for distribution of TAMs, Atanasov et al. concluded that a high density of TAMs predicts high recurrence and poor prognosis of hilar CCA.^81^ A wide array of studies showed that experimental and presumably human CCA is a Wnt‐driven tumour,^82^, ^83^, ^84^ and the Wnt signalling pathway can be activated by TAMs, offering a potential therapeutic target for CCA.^9^, ^11^, ^85^

#### 3.5.2 Endothelial cells

Performed with in vivo murine and human tumour models, Cao et al. verify that the loss of insulin growth factor binding protein‐7 expressed by tumour associated endothelial cells (TAEs) induces the tumourigenesis and invasion of tumours through activating FGF4‐FGFR1‐ E26 transformation specific (ETS)2 pathway.^86^ TAEs are also associated with tumour angiogenesis. Co‐elevated VEGF and syndecan‐1 in tumour stroma enhance expressions of integrin and VEGF receptors on TAEs to stimulate the angiogenesis of tumors.^87^ Endothelial cells can release inflammatory chemokines to attract leukocytes to establish a pro‐fibrotic and pro‐angiogenic microenvironment and promote the migration, invasion and epithelial‐mesenchymal transformation (EMT) in CCA.^88^ However, endostatin precursor secreted by CCA stromal endothelial cells suppresses tumour angiogenesis, growth, and development.^89^ Weakening of the adhesion between CCA cells and vasal endothelial cells results in a suppression of the metastasis of CCA cells. CCA associated endothelial cells highly express erythropoietin receptor (EpoR) that bind to liver‐cell‐released erythropoietin (Epo) in the tumour microenvironment, thus promoting proliferation, survival, and invasion of CCA cells.^90^

#### 3.5.3 Mast cells

Mast cells possess basophilic granules that contain heparin, 5‐hydroxytryptamine, histamines, and cytokines, which inhibits blood coagulation, suppresses nerve conduction, increases vascular permeability, and enhances active immunity, respectively.^91^ In addition, mast cells promote immunoregulation and stimulate immunocytes (eg macrophages, DCs, T lymphocytes, B lymphocytes, eosinophil granulocytes, and NK cells) in immune responses through mediating the secretion of inflammatory factors and enzymes. And mast cells take a significant role in inflammation‐mediated tumourigenesis (eg inflammation‐mediated colorectal tumourigenesis).^92^ Mast cells are present at not only normal connective tissue but also CCA tissue, and the amount of mast cells increase during CCA carcinogenesis that exerts significant influence in CCA growth. Mast cells recruited in the CCA microenvironment via c‐Kit/stem cell factor in response to inflammation can release histamine to accelerate CCA growth and angiogenesis.^93^

#### 3.5.4 T lymphocytes

Generation, activation, differentiation, and functions of T lymphocytes are affected by cellular metabolism related to cancer surveillance and inflammatory disorders. T lymphocytes are quite complex and heterogeneous, and they are constantly updated in vivo and can exist at different developmental stages or functional subgroups at the same time. T helper cells are categorized as Th1 cells that are involved in cell mediated immunity and tumour suppression by secreting cytokines and chemokines like INF‐γ, Th2 cells that regulate humoral immunity, and Th17 cells.^94^ Cytotoxic T lymphocytes can program the death of cancer cells by releasing granzyme serine proteases A and B and a pore‐forming protein.^95^ However, CD4(+)Foxp3(+) regulatory T cells accelerate the death of NKs and CD8+ T lymphocytes to inhibit T cell‐mediated anti‐tumour effect via utilizing granzyme B and perforin.^96^ By comparing the density of T regulatory cell density and S100A9+ cells linked to immunosuppression in areas of tumour progression and tumour regression, Gray et al. proved that T lymphocytes are important players in tumour progression and regression by regulating immune response.^97^ Previous scientific studies have shown that CD8+ T lymphocytes are present in several cancers and are associated with cancer prognosis. In the case of CCA, CD8+ T lymphocytes have been shown to exist in the tumour microenvironment.^98^, ^99^ Kim et al. further demonstrated that CD8+ T lymphocytes were present in CCA. And it is not CD8+ T lymphocytes but memory type CD8+ T lymphocytes (CD8+CD45RO+) that are important for the prognosis of CCA.^100^ Besides, programmed cell death ligand 1 (PD‐L1), a protein that can be expressed on CCA cells^100^ and the stromal cells of CCA, is highly expressed in the area where there are abundant tumour infiltrating T lymphocytes in CCA.^101^ High expression of PD‐L1 is associated with poor prognosis of CCA which support the potential rationale of using PD‐1 blockade immunotherapy in CCA.^102^, ^103^ As a portion of tumour stromal cells, different types of T lymphocytes have different effects on tumour progression including suppressing tumourigenesis and inhibiting anti‐cancer immune reactions.

### 3.6 Epithelial cells and mesenchymal stem cells

Metastasis, the main characteristic of carcinoma, is a major factor deciding the cancer‐associated mortality in patients and related to EMT whose main participators are epithelial cells and MSCs. Epithelial cell extrusion involving the sphingosine‐1‐phosphate ‐ sphingosine‐1‐phosphate receptor 2 pathway enables the detachment of tumour cells from their primary sites, prompting the metastatic process.^104^ MSCs recruited from the bone marrow and adjacent tissue by cytokines and chemokines are integrated into tumour stroma, and then become tumour‐derived MSCs modulating tumourigenesis and tumour progression.^105^ MSCs include two distinct phenotypes, MSC1 and MSC2, with different effects on cancer cells. MSC2 enhances tumour growth and metastasis while MSC1 does not.^106^ MSCs can enhance tumour progression via the VEGF pathway and the secretion of CCL5, but their effects on tumour vary under different conditions.^107^, ^108^ For example, they promote the growth of breast cancer through the WNT signalling.^109^ In a xenograft ovarian carcinoma model, Spaeth et al. discovered MSCs have multipotential capacity to differentiate into carcinoma associated fibroblasts that contribute to tumour progression and angiogenesis.^110^ EMT induces the epithelial cells to undergo morphological changes, gives the cell metastasis and invasion ability and relates to the formation of circulating tumour cells which contributes to the tumour invasion and metastasis.^111^ CCA is a malignancy that occurs in epithelium and in some cases the proliferation of bile duct epithelial cells accelerates CCA cells proliferation by overexpressing synemin (an intermediate filament protein in vascular cells and hepatic stellate cells, is over expressed in inflammation and fibrosis).^112^ On the other hand, epithelial cells can occur EMT under the influence of miR‐21 and Kruppel‐like factor 4 via the Akt and extracellular signal‐regulated kinase (ERK) 1/2 pathway, thus impacting the migration and growth of CCA.^113^ Additionally, EMT augments the invasion and metastasis of CCA cells that expedites the progression of carcinoma^114^, ^115^ and it can be induced and adjusted by atypical protein kinase C‐iota (aPKC‐ι),^116^ EGF/EGFR axis^27^ and protein tyrosine phosphatase PTP4A1.^117^

### 3.7 Hepatic stellate cells

HSCs are in Disse separation and glued to liver sinusoidal endothelial cells. During fibrosis and cirrhosis, HSCs can differentiate into myofibroblasts,^118^ and the latter has been proven a crucial player in promoting CCA growth and progression via releasing α‐SMA and EGFR signalling.^119^ HSCs can be activated by numerous factors (cytokines and chemokines) and signalling pathways,^32^ including NF‐κ B, TGF‐β, PDGF, and Hedgehog signalling that translates signal between CCA cells and HSCs.^120^ Clonorchis sinensis can produce protein complex (clonorchis sinensis ferritin heavy chain CsFHC) to activate hepatic stellate cells in hepatic fibrosis and inflammation^121^, ^122^ and accelerate CCA through relevant mediators.^123^ What's more, HSCs can increase the expression of CXCL5 in CCA cells by secreting IL‐β to enhance the connection between CCA cells and CAFs that influences the progression of CCA.^124^ The interaction between HSCs and Angiotensin (Ang) II induces tumour proliferation via Ang II/Ang II type 1 receptor (AT‐1) axis.^125^

### 3.8 Cancer stem cells

Cancer stem cells (CSCs) are a group of cells that have the ability of self‐renewal, hyperproliferation, and differentiating into tumour cells. It has already been confirmed that the existence of CSCs in CCA.^126^ Laminin‐332, exists in CCA CSCs matrix, builds a circumstance of chemoresistance and quiescence for CCA.^127^ Low expression of CD274 in CCA cells can influence the interaction between CSCs and tumour cells to increase tumourigenesis and help cancer cells escape from the immune reaction.^128^ CCA associated stem cells have a strong relation with the initiation and development of CCA.

## 4 FUTURE DIRECTION AND OUTLOOK

Malignant potential of CCA is not only a consequence of cell transformation, but also a result of a complicated and changeable interaction between cancer cells and supporting stroma in tumour microenvironment. And numerous facts verify tumour microenvironment play an important role in CCA invasion, proliferation, and progression. Exploring and understanding the interaction between tumour cells and tumour microenvironment will provide innovative and precise anti‐CCA therapies for patients. From the above introduction of CCA microenvironment, it can be seen that hypoxia, exosomes, inflammatory cytokines, CCA associated fibroblasts, inflammatory cells, epithelial cells, MSCs, HSCs, and CSCs play their different roles in CCA progression which may be attractive targets for CCA therapy. Mainly, the treatment methods of microenvironment orientation of CCA could aim at disturbing the signalling pathways of tumourigenesis in CCA cells which induced by CCA microenvironment, blocking the function of tumour‐derived cytokines and chemokines which could recruit activated stromal cells to the CCA tumourigenic region and inducing the apoptosis of CCA stromal cells which could promote the invasion, angiogenesis, metastasis, and growth of CCA cells. In accordance to previous research, Hu MH and colleagues have verified that SC‐43 (a sorafenib derivative) induced the apoptosis and death of CCA cells through enhancing SH2 domain‐containing phosphatase 1 (SHP‐1) activity which leads to phosphorylation STAT3 and downregulates cyclin B1 and Cdc2.^129^ TAMs promote CCA angiogenesis and assist extracellular matrix decomposition and reconstruction to enhance tumour invasion and growth by expressing MMP‐9 and communicating with CCA cells. Thus, TAMs are at the centre of the invasion microenvironment and are a crucial and creative drug target for cancer therapy.^80^ Similarly, other tumour stromal cells in CCA microenvironment can become drug target for cancer therapy, too. More comprehensive and complete mechanisms of the interaction between CCA and tumour microenvironment need to be explored and perfected and it will be the basis for our search for a more effective and accurate treatment for CCA. New treatment regimens combined with old treatments (Surgical resection, chemotherapy, radiotherapy, and transplantation therapy) will offer new hope for the treatment of CCA.

## CONFLICTS OF INTEREST

The authors confirm that there are no conflicts of interest.
